# Protocol of the Belgian food consumption survey 2014: objectives, design and methods

**DOI:** 10.1186/s13690-016-0131-2

**Published:** 2016-05-16

**Authors:** Sarah Bel, Sofie Van den Abeele, Thérésa Lebacq, Cloë Ost, Loes Brocatus, Charlotte Stiévenart, Eveline Teppers, Jean Tafforeau, Koenraad Cuypers

**Affiliations:** Scientific Institute of Public Health, Department of Public Health and Surveillance, Unit Surveys, Lifestyle and Chronic Diseases, Brussels, Belgium

**Keywords:** Food consumption survey, Habitual intake, Dietary monitoring, 24-hour recall, Food safety, Food frequency questionnaire, Physical activity

## Abstract

**Background:**

Dietary patterns are one of the major determinants as far as health and burden of disease is concerned. Food consumption data are essential to evaluate and develop nutrition and food safety policies. The last national food consumption survey in Belgium took place in 2004 among the Belgian population aged 15 years and older. Since dietary habits are prone to change over time a new Belgian National Food Consumption Survey (BNFCS2014) was conducted in 2014–2015.

**Methods:**

The BNFCS2014 is a cross-sectional study. A representative sample (*n* = 3200) of the Belgian population aged 3 to 64 years old was randomly selected from the National Population Register following a multistage stratified sampling procedure. Data collection was divided equally over the four seasons and days of the week in order to incorporate seasonal effects and day-to-day variation in food intake. Information on food intake was collected in adults with two non-consecutive 24-h dietary recalls (using the GloboDiet® software). In children food intake was collected with two non-consecutive one-day food diaries followed by a completion interview with GloboDiet. Additional data on socio-demographic characteristics, eating habits, lifestyle, food safety (at household level), physical activity and sedentary behaviour were collected with a face-to-face questionnaire using a computer-assisted personal interviewing technique. In the time between the two visits, participants were asked to complete a self-administered food frequency questionnaire and health questionnaire. Height, weight and waist circumference were measured. In addition, children and adolescents were asked to wear an accelerometer and keep a logbook for seven consecutive days to objectively measure physical activity and sedentary behaviour.

**Conclusion:**

The main objective of the BNFCS2014 is to evaluate the habitual food, energy and nutrient intake in the Belgian population and to compare these with recommendations from the national dietary guidelines. A second objective is to monitor eating habits and food safety aspects of the food consumption in Belgium. The results of this dietary monitoring survey, together with the information on the level of physical activity, may underpin future nutrition, food safety and physical activity policies at national and European level.

## Background

Non-communicable diseases (NCDs), including ischemic heart disease, stroke and diabetes mellitus, are the leading causes of death and disease burden globally [1, 2]. Dietary risk factors and physical inactivity are considered as one of the main risk factors for global disease burden [3]. In Belgium dietary risks account for the most disease burden, followed by smoking and high body mass index (BMI) [4]. To prevent and control NCDs there is a need to develop and implement effective nutrition and health policies together with nutrition education programmes addressing these modifiable risk factors [5]. In this context food consumption data collected at an individual level are essential [6]. Regular dietary monitoring with a description of a) the food consumption, b) the intake of energy, macronutrients and micronutrients, c) the trends in dietary behaviour, d) anthropometric indicators (e.g. weight) and related health outcomes of a population forms the basis of nutrition and food safety policy [7]. Accurate and detailed food consumption data are also necessary for the assessment of exposure to potentially hazardous substances [8]. Furthermore, food consumption surveys provide information that is needed for scientific research on associations between nutrition and health.

The first nationwide individual dietary survey conducted in Belgium was the Belgian Interuniversity Research on Nutrition and Health (BIRNH) organised in the early 1980s with a total of 11000 participants aged 25 to 74 years [9]. The main objective of the BIRNH was to study the association between nutrition and total and cause-specific mortality. The results showed that the macronutrient intake deviated from national dietary guidelines [10]. In 2004 the first national food consumption survey was conducted in Belgium. Information on dietary intake was obtained from 3245 individuals aged 15 years and older [11]. Food intakes differed substantially from the food-based dietary guidelines [12] and dietary macronutrient intakes deviated from the national dietary guidelines and differed between age classes [13]. Based on these results, efforts have been taken to improve the Belgian food pattern with programs such as the Belgian National Food and Health Plan (2005–2010) (BNFHP) [14]. Since food consumption patterns are subject to changes, recent information on food consumption is required. In addition, nutrition and food safety policies need to be evaluated. This is especially needed for children younger than 15 years old; they were not included in the previous surveys and as a consequence no national food consumption data is available yet for this age group. Infants and young children are considered the most vulnerable, thus taking priority for exposure risk assessments [6]. Therefore, in 2014–2015 a second Belgian national food consumption survey (BNFCS2014) was conducted which was initiated by the Belgian Federal Public Service Health, Food Chain Safety and Environment. The study was carried out by the unit Surveys, Lifestyle and Chronic Diseases of the Scientific Institute of Public Health.

The BNFCS2014 also plays part in the European Union (EU) Menu project, a pan-European food consumption survey. This project is coordinated by the European Food Safety Authority (EFSA) and aims at harmonising data collection on food consumption across the EU member states. The availability of comparable food consumption data across the EU is crucial in order to efficiently assess the accurate exposure of the population to contaminants. It also supports European policy makers in the assessment of the nutritional status of population subgroups, setting targets regarding healthy diets and monitoring progress over time [6].

The BNFCS2014 will result in a large amount of valuable data for which many articles and reports are expected. This paper describes the objectives, design and methods of the BNFCS2014.

## Study objectives

The BNFCS2014 has the following specific objectives:

i. to evaluate the habitual food, energy and nutrient intake in the general Belgian population (including children) and to compare these with recommendations from the national dietary guidelines;
ii. to assess the use of dietary supplements and the intake of micronutrients from dietary supplements;
iii. to investigate the difference in food, energy and nutrient intake between different subgroups of the population and to identify subgroups at risk for a deficient or excessive intake of specific foods or nutrients;
iv. to monitor food safety aspects of the food consumption in Belgium through evaluating food safety practices and providing data for estimating the intake of contaminants, additives and other chemicals in food;
v. to evaluate the physical activity and sedentary behaviour of the general Belgian population and use this information in relation to food and nutrient intake.

The BNFCS2014 also serves as a follow-up study by comparing results with those of the previous food consumption survey carried out in 2004.

## Methods

The methodology of the BNFCS2014 followed to a large extent the guidelines published by EFSA in view of the EU Menu project [6, 8] as well as the recommendations made after the Pilot Study for the Assessment of Nutrient intake and food Consumption Among Kids in Europe (PANCAKE) project [15]. Approval of an Ethical Committee (University of Ghent) and the Commission for the Protection of Privacy were obtained. The study was conducted in accordance with the ethical principles for medical research involving human subjects (Declaration of Helsinki).

### Sampling design

The target population was defined as persons aged 3 to 64 years old with their residence in Belgium. The National Population Register (NPR) was used as the sampling frame; as a consequence, persons not officially registered (homeless people, unofficial immigrants, etc.) were excluded a priori from the study. Selected persons could be excluded a posteriori during the recruitment if they were found to be ineligible: a) institutionalized people (because of a reduced freedom in food choice), b) individuals residing abroad or who moved out of the selected municipality, c) individuals not able to be interviewed because of a physical or mental disability and d) individuals unable to speak Dutch or French [8]. The sample selection was performed in seasonal waves (4 samples) in order to use the most actual version of the NPR to ensure the compatibility of age groups with the target population and avoid subjects who had moved between the date of selection and the date of interview.

EFSA recommends to collect food consumption data in specific age groups, including children (3–9 years), adolescents (10–17 years) and adults (18–64 years) [6]. Taking this into account, the sample was stratified in ten age-gender strata allowing the description of food consumption in five age groups (3–5, 6–9, 10–17, 18–39 and 40–64 years) and both genders.

The sample size was defined based on sample size calculations (using the three age groups proposed by EFSA), but was ultimately also determined by budget constraints and available logistics means. The sample size was calculated in order that the estimated mean intake for different nutrients falls within a 5 % interval around the true population mean with a 95 % probability. It was decided to include 1000 children aged 3 to 9 years old, 1000 adolescents aged 10 to 17 years old and 1200 adults aged 18 to 64 years old, resulting in a total sample size of 3200 participants, which is similar to the sample size of the previous survey [11]. This sample size exceeds the recommendation of 260 children (3–9 years), 260 adolescents (10–17 years) and 260 adults (18–64 years) made by EFSA [6].

**Fig. 1:**
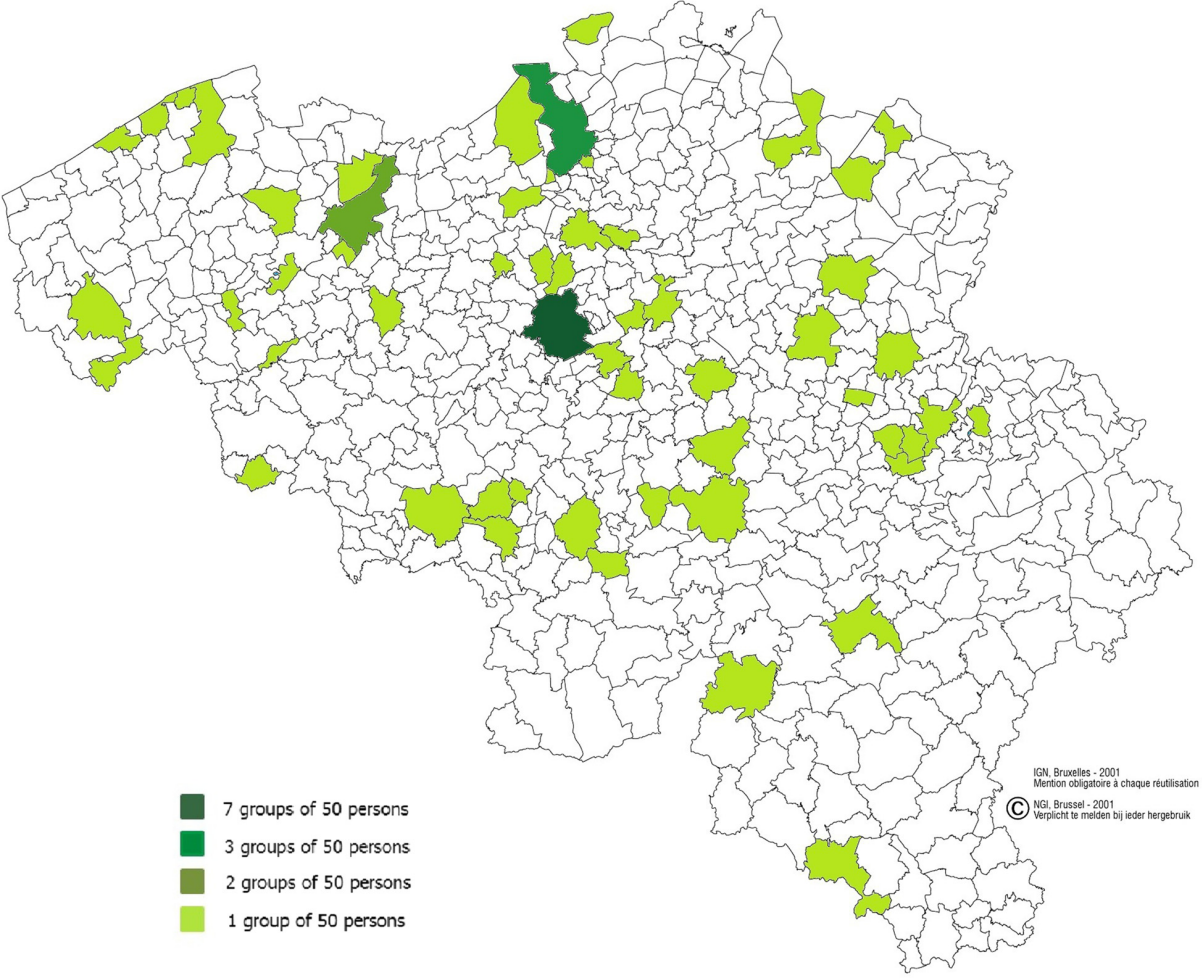
Selected municipalities in the BNFCS2014

The participants of the BNFCS2014 were selected according to a multistage stratified sampling procedure, including a geographical stratification, a selection of municipalities within each geographical stratum (i.e. the primary sampling units (PSUs)) and a selection of individuals within each municipality (i.e. the secondary sampling units (SSUs)). This sampling scheme is very similar to the one used in the Belgian Health Interview Surveys [16]. The 3200 interviews for the whole of Belgium were thus divided in eleven provincial strata (Antwerp, Flemish Brabant, West Flanders, East Flanders, Limburg, Brussels-Capital region, Walloon Brabant, Hainaut, Liège, Luxembourg, and Namur). The number of interviews to be realized within each province was proportional to the population size of each province. A clustered selection procedure was chosen where the number of interviews to be realized within each province was divided by 50 to define the number of municipalities (PSUs) that had to be selected. The municipalities were selected within each stratum based on a method that combines probability proportional to size sampling and systematic sampling. This strategy ensures that big cities as well as small municipalities could be selected for the survey. The geographical dispersion of the selected municipalities for the BNFCS2014 is presented in Fig. 1. The figure also shows in which municipalities more than one PSU were selected. In the last step, individuals or SSUs were randomly selected within each PSU and age-gender stratum.

As the participation to the BNFCS2014 is voluntary, it was expected that some of the selected individuals would not participate either because we would not be able to contact them or because they would refuse to participate. Therefore a matched substitution was applied: for every selected individual three consecutive individuals were selected as substitute. The selected individual together with its substitutes were considered as one cluster and had the following characteristics in common: municipality of residence, gender and age. If the first individual in the cluster refused participation, the next individual in the cluster was contacted. This continued until the cluster was exhausted. The number of clusters in every PSU was doubled to ensure that the predetermined number of interviews could be achieved. The individuals of the initial and substitute cluster had the following characteristics in common: municipality of residence, gender and age stratum.

### Recruitment

Participants were invited by an invitation letter together with an information brochure. These documents informed the selected individuals about the objectives and content of the BNFCS2014. Within two weeks after reception of these documents the selected individuals were contacted by the interviewer; this first contact (either by phone or at the doorstep) was the opportunity to explain further the study, determine the eligibility of the selected person and obtain consent for participation. At least five contact attempts had to be made (either a home visit or by phone) for each selected individual before they were classified by the interviewer as non-contactable. If a selected individual was contactable but refused participation, the reason for non-participation was noted. Written informed consent was obtained from the participants or the parent(s)/legal guardian(s) of participants younger than 12 years old, before the start of the first home visit.

### Study design

The BNFCS2014 is a cross-sectional study. The data collection was divided equally over the four seasons and all days of the week in order to incorporate seasonal effects and day-to-day variation in food intake. The interviewers collected information from the participants by a face-to-face interview during two home visits with a 2 to 4 week interval. In children (aged 3 to 9 years old) a parent or legal guardian was used as a proxy respondent. Table 1 gives an overview of the data collection methods used in the BNFCS2014 and the differences in collection methods between children, adolescents and adults.

**Table 1:** Overview data collection methods of BNFCS2014 in children and adolescents/adults

Age group	First home visit	Between home visits	Second home visit
Children (3–9 years)	• Face-to-face questionnaire^a^(CAPI)	• 1-day food diary + GloboDiet completion interview 1 by telephone • FFQ (paper) • Health questionnaire (paper) • Accelerometer + logbook • 1-day food diary (for completion interview 2)	• GloboDiet completion interview 2 • Anthropometric measurements
Adolescents and adults (10–64 years)	• 24-h recall 1 (GloboDiet) • Face-to-face questionnaire^a^(CAPI) (part 1)	• FFQ (paper) • Health questionnaire (paper) • Accelerometer + logbook (adolescents)	• 24-h recall 2 (GloboDiet) • Face-to-face questionnaire^a^(CAPI) (part 2) • Anthropometric measurements

*CAPI* computer-assisted personal interviewing, *FFQ* Food Frequency Questionnaire

^a^The face-to-face questionnaire includes questions regarding physical activity, sedentary behaviour, socio-demographic, lifestyle and health characteristics and food safety

During the first home visit the interviewer performed a) a 24-h recall to assess food intake and b) a face-to-face interview on background, health and behavioural characteristics. In the time between the two visits, participants were asked to a) complete a food frequency questionnaire (FFQ), b) fill in a self-administered questionnaire about health, c) wear an accelerometer and fill in a logbook on physical activity (children and adolescents). During the follow-up home visit a second 24-h recall was performed and anthropometric measurements were taken.

A computer-assisted 24-h recall has been used to assess the dietary intake of the previous day in adolescents and adults. To collect dietary intake in children, a parent (or legal guardian) was asked to keep a food diary on two non-consecutive days. This was followed by a completion interview with a computerised 24-h recall program. The first completion interview was performed by telephone, or exceptionally by an additional face-to-face interview, before the follow-up home visit. During the follow-up home visit a second 24-h recall was performed in adults and adolescents. In children the second completion interview based on the completed one-day food diary was performed.

Additional data on socio-demographic characteristics, eating habits, lifestyle, food safety and physical activity were collected with a face-to-face questionnaire using a computer-assisted personal interviewing (CAPI) technique. The interviewer asked questions, while showing the possible answer categories to the respondent or proxy respondent on a card. The answers were directly entered into a portable computer. The CAPI technique enhances the quality of collected data by including automatic jumps and the ability to spot inconsistent or impossible answers. Moreover, it makes the process of post-collection data entry, a possible source of error, unnecessary.

Accelerometers were used to measure physical activity and sedentary behaviour during seven consecutive days; this was complemented by filling in a logbook to note the time and reason for removing the accelerometer.

The following anthropometric measurements were taken: weight, height and waist circumference.

Finally, the interviewer collected the questionnaire on health (during the second home visit) in a sealed envelope because of the sensitivity of questions.

### Data collection

#### Dietary assessment

##### 24-h dietary recall

Dietary assessment in adolescents and adults was performed by the 24-h dietary recall method, carried out on two non-consecutive days, as recommended by EFSA [8]. The interviewer asked the respondent to reproduce the foods and beverages (and dietary supplements) consumed in the preceding day, including their quantity [17]. A single 24-h recall is not suitable for determining distributions of usual dietary intake due to within-subject variation. At least two independent days are needed to apply statistical modelling to estimate habitual intake [18]. The time between the two non-consecutive 24-h recalls should be at least one to two weeks as this provides a better estimate of within-subject variation [6]. The validity of two non-consecutive 24-h recalls to assess habitual food intake among the Belgian population aged 15 years and older was confirmed in a previous study by comparison with 5-day estimated dietary records [19].

GloboDiet (former EPIC-Soft) is a computerised 24-h recall program that has been developed and is maintained by the International Agency for Research on Cancer (IARC). Due to the high level of standardisation and international use, GloboDiet is the most appropriate dietary software which enables the collection of dietary data within a pan-European survey [6]. The use of the GloboDiet methodology in epidemiological studies and pan-European food consumption surveys has been tested in several projects, such as the European Prospective Investigation into Cancer and Nutrition (EPIC) study, the European Food Consumption Survey Method (EFCOSUM) project and the European Food Consumption Validation (EFCOVAL) project [20]. GloboDiet systematically follows standardised steps when describing, quantifying, probing and calculating food intake [20]. GloboDiet was also used in the previous national food consumption survey of 2004 [8]. In comparison with the previously used version a number of new specifications and facilities were implemented to the program [21]. A new country-specific GloboDiet version was prepared for this study (EPIC-Soft version 0.2014.02.10).

The 24-h recall interview performed with GloboDiet is divided into five main steps: (1) general non-dietary information; (2) quick list, i.e. a chronological list of consumed foods and recipes by eating occasion; (3) description and quantification of foods and recipes; (4) dietary recall summary and final checks (quality check at nutrient level) and (5) dietary supplement description and quantification [21]. Food portion sizes were quantified using household measures (e.g. glasses, cups, spoons, etc.), food portions (obtained from manufacturer’s information) and a picture book. The picture book included a selection of pictures of country-specific dishes from the EPIC-Soft picture book [22], the PANCAKE study picture book (validated in children of 0–10 years of age) [23], the Swiss picture book [24] and the French picture book for the national Survey on Food Consumption (INCA3) [25]. The picture book also included drawings of bread, representing the actual shape and size of bread slices. These drawings were developed for use in the previous Belgian food consumption survey from 2004 and their use for portion size estimation of bread in nutrition surveys was found to be acceptable [26].

Although GloboDiet involves a structured and standardized approach, trained and experienced interviewers are needed to ensure good quality of the open-ended interview. It is very important that interviewers have thorough knowledge of the available foods on the market and the recipes usually prepared within the study population [27]. Therefore, in the BNFCS2014 only dieticians were used as interviewers.

Dietary assessment in children (3 to 9 years old) was done using two self-administered non-consecutive one-day food diaries followed by a GloboDiet completion interview with the proxy respondent: one by telephone (after the first home visit) and one face-to-face at the respondent’s home (during the second home visit). The one-day food diaries had to be completed one or maximum two days before the completion interview with Globodiet. The PANCAKE project concluded that this approach offers the most advantages for the future pan-European survey in children aged 0 to 10 years compared to other dietary intake methods [15]. All food-diaries were open-ended (i.e. no pre-coded food lists) and special pages were available for home-made recipes and dietary supplement intake. In every booklet explanations and examples on how to fill in the diary were provided.

##### Food frequency questionnaire

As recommended by EFSA [6], an age-appropriate self-administered food frequency questionnaire (FFQ) was used in all age groups to ask the respondents to report their usual frequency of consumption of specific foods and dietary supplements in the last 12 months. The main objective of the FFQ was to identify never-consumers (persons who indicate they never consume a specific food or dietary supplement) for usual intake modelling purposes [6, 18]. A list of 79 food items (74 food items in children excluding alcoholic beverages) was used, based on the food list of the FFQ used in the previous food consumption survey in 2004 [11]. This self-administered qualitative FFQ was found to be reasonably valid in both genders and across different age categories for most food groups. However, a rather low relative validity was found for the food groups bread and cereals, potatoes and grains, and sauces [28]. As some food items were added or reformulated, the validity of the used FFQ should be re-evaluated. The frequency response options for the food list were: never, less than once per month, 1–3 times per month, once per week, 2–4 times per week, 5–6 times per week, once per day, 2–3 times per day and more than 3 times per day.

For the dietary supplements the frequency options ‘2–3 times per day’ and ‘more than 3 times per day’ were grouped together as ‘once per day’. The questionnaire distinguished the use of dietary supplements during wintertime and during the rest of the year.

##### Data analysis

The detailed food consumption data collected with GloboDiet has to be complemented with detailed information on the nutrient concentration of each specific food item. This is obtained by linking the survey data with a national food composition database (the Belgian Food Composition Data NUBEL including branded foods [29]) providing detailed information on the nutritional composition of foods. In addition the Dutch Food Composition data, NEVO, was used for foods that were not included in the national food composition database or contained more complete information on nutrient concentrations [30]. All foods recorded in the survey are also classified in accordance with the FoodEx2 food classification system developed by EFSA for harmonising pan-European exposure assessments [6, 31].

The repeated 24-h recall data in combination with the FFQ data and statistical models can be used to estimate the habitual intake distribution by statistical correction for within-subject variation [18, 32]. The Statistical Program to Assess Dietary Exposure (SPADE) is used to estimate the habitual intake distribution of dietary components (i.e. macronutrients and micronutrients) and foods [33]. SPADE is a R package, freely available, which includes several modelling options. Four option models can be used for the analysis. The first option models dietary components and foods that are consumed on a daily basis by almost all participants (1-part model). The second option concerns dietary components and foods that are consumed episodically (2-part model). If information on never-consumers is available for a specific food or dietary supplement (from the FFQ) it can be considered in this model. The third model is specifically aimed to estimate total nutrient intake from foods and dietary supplements (3-part model). With the fourth option, the habitual intake of nutrients from multiple food sources or food groups can be estimated in a multi-part model. For all of these models, the habitual intake distribution is modelled as a function of age, and this distribution can be directly compared with cut-off values (e.g. estimated average requirement, adequate intake and tolerable upper intake level) to estimate the proportion above or below. Uncertainty in the habitual intake distribution and in the proportion below or above a cut-off value is quantified with ready-for-use bootstrap and provides confidence intervals with the required confidence level [33].

#### Physical activity and sedentary behaviour

Physical activity (PA) and sedentary behaviour (SB) were measured by using a self-report PA questionnaire (using the CAPI technique) and accelerometers.

Different self-report questionnaires were used according to the age of the respondents. In adults the International Physical Activity Questionnaire long form (IPAQ-LF) was used to assess physical activity levels. This questionnaire has been extensively validated in previous studies [34]. In adolescents the Flemish Physical Activity Questionnaire (FPAQ) was used, which has been validated in Flemish adolescents between 12 and 18 years of age [35]. In children the proxy-report questions regarding PA and SB used in the TOYBOX cross-sectional study, a European study with the aim to collect information on the prevalence of overweight and obesity, and on energy balance-related behaviours (including PA and SB) in children aged 4–6 years, were used [36].

Because the validity of self-reported PA questionnaires is considered poor to moderate, especially in children and adolescents [37], an objective method for assessing PA was included. This is an improvement in comparison with the previous survey in 2004 which only used self-reported information. Accelerometers provide objective and accurate measurement of body accelerations, which are expressed as ‘counts’ and can be summed over a specific time interval or ‘epoch’. These counts values can be converted into time spent in a specific activity category, ranging in intensity from sedentary to very vigorous.

Children (3 to 9 years old) and adolescents (10 to 17 years old) wore Actigraph GT3X+ triaxial accelerometers attached to an adjustable elastic waist belt on the right hip during 7 consecutive days. During this period, a logbook was also kept to keep track of the time of getting up in the morning and going to bed for sleeping. Also the time and reason for removing the device for more than five minutes for activities such as swimming (as water-based activities cannot be measured) had to be noted in the logbook.

Accelerometers were initialized using Actilife software [38] and an epoch length of 15 s was selected [39]. The software Meterplus 4.3 is used to screen, clean and score the accelerometer data files [40]. Non-wearing time is calculated as periods of more than 20 min of consecutive zero counts. Participants are included if they had at least 2 weekdays with minimum 10-h wearing time and 1 weekend day with minimum 8-h wearing time. Moderate-to-vigorous physical activity (MVPA) and sedentary time in children and adolescents are calculated using the activity cut-points of Evenson et al. [41].

#### Socio-demographic, lifestyle and health characteristics

A face-to-face interview (CAPI) with the (proxy) respondent obtained socio-demographic information on all the members of the household: relationship to the selected person, age, gender and educational attainment (coded according to the International Standard Classification of Education 2011 levels of education classification [42]). The occupational class of the participant or parent(s), classified according to the Erikson Goldtorpe Portocarero social class scheme [43], was also asked.

Additional information was obtained from the selected person regarding: eating habits (e.g. preparation and consumption times of meals, family meals), pregnancy status, breastfeeding, smoking behaviour, use of (iodized) salt, use of vitamin D supplements, parental feeding practices (only in children), meal frequency, consumption of organic products and opinion on Belgian nutrition policies.

In adolescents and adults a self-administered paper and pencil questionnaire collected information regarding several health characteristics: eating disorders using the 7-item version of the Eating Attitude Test [44], self-perceived health using a one-question instrument proposed by the World Health Organization [45], psychological distress using the 5-item version of the Hopkins Symptom Checklist [46], weight management behaviour and prevalence of a list of five nutrition related diseases (diabetes, hypertension, hypercholesterolemia, cardiovascular diseases and food allergy). In adolescents this questionnaire additionally collected information regarding smoking behaviour and pubertal development using the Pubertal Development Scale [47]. In children only information regarding self-perceived health and nutrition related diseases was obtained. As all these questions were considered to be sensitive, they were not included in the face-to-face interview and collected in a sealed envelope by the interviewer.

#### Food safety

The knowledge, attitude and behaviour of the population on food safety and hygiene (e.g. taking into account food expiration dates, safe food handling practices, etc.) were collected at the household level. The face-to-face questions (CAPI) regarding food safety had to be answered by the member of the household of the participant who is usually involved in the preparation of meals.

#### Anthropometric measurements

Anthropometric measurements (weight, height and waist circumference) were gathered by the trained interviewers following a standardized protocol. In contrary to the previous survey weight and height were gathered by actual measurements instead of self-reporting. Participants were measured in light clothing and without shoes. The body weight was measured to the nearest 0.1 kg using an electronic scale (type SECA 815 or SECA 804). The height was measured to the nearest 0.5 cm with a portable stadiometer (Type SECA 213) with the head positioned in the Frankfort horizontal plane. The waist circumference was measured to the nearest 0.5 cm with a non-stretchable measuring tape (type Meterex) midway between the lower rib and the iliac crest.

Height and weight are used to calculate the BMI in order to assess the prevalence of overweight and obesity using age-appropriate cut-off values. Height and weight (together with age and gender) are also used to estimate the basal metabolic rate (BMR) to identify under-reporters of energy intake (EI). The ratio of reported daily EI divided by the estimated BMR can be compared with the Goldberg cut-off for plausible EI (taking into account the body weight stable assumption) [48]. Waist circumference is used to measure abdominal adiposity using age-and gender-appropriate cut-off values and to calculate the waist-to-height ratio.

### Interviewers

All interviews were carried out by full-time or part-time independent dieticians with sufficient social and computer skills. They received a three-day training. On the first day information about their tasks as interviewer was given, followed by practicing the instruments used during the interview. On the second and third day the interviewers received a specific training regarding GloboDiet. Interviewers could start the fieldwork after approval of case study interviews made at home.

To ensure the quality of the 24-h recalls the first ten interviews of each interviewer were immediately evaluated with feedback to the interviewer. Furthermore, regular newsletters and individual feedback were sent during the whole data collection period to the interviewers to clarify points of interest raised during the course of the fieldwork. All the procedures of the fieldwork and GloboDiet were described in exhaustive training manuals for the interviewers.

## Discussion

The study methodology of the BNFCS2014 is quite comparable with that of the first food consumption survey carried out in 2004 [11]. However, differences exist due to the fact that, contrary to the previous study, children younger than 15 years are included in the BNFCS2014, demanding an appropriate methodological approach. Similar to the previous study, information on food and dietary supplement intake in adolescents and adults was collected using two non-consecutive 24-h dietary recalls. Dietary assessment in children was however performed differently, using two non-consecutive one-day food diaries followed by a completion interview. Both assessment methods were combined with an age-appropriate self-administered FFQ.

A lot of questions were adopted from the 2004 survey to ensure comparability, but several new questions were also added (e.g. parental feeding practices, family meal frequency and pubertal timing).

The BNFCS2014 was able to make some methodological improvements in comparison with 2004. The previous study used a self-report PA questionnaire (IPAQ short form) to assess PA. However, such assessments based on self-report may not accurately reflect habitual activity patterns due to recall bias and/or reporting bias caused by social desirability [49]. Moreover, there is currently no PA questionnaire for children or adolescents with an acceptable validity and reliability [50]. Therefore, in this survey PA an SB was, next to age-appropriate self-report questionnaires, also objectively measured using accelerometers in children and adolescents.

In contrary to the previous survey, this study gathers all anthropometric measurements (weight, height and waist circumference) by actual standardized measurement procedures. Self-reports have been found to underestimate weight and overestimate height, therefore underestimating the prevalence of overweight and obesity [51].

Another strength of the BNFCS2014 is the inclusion of children younger than 15 years old. The monitoring of food consumption in children is important since major and increasing public health problems such as obesity and diabetes are related to dietary habits and lifestyle factors during childhood. In addition, children are considered as a vulnerable age group with respect to food safety.

Finally, the creation of a database providing detailed information on the nutritional composition of dietary supplements allows this study, contrary to the previous one, to calculate the contribution of dietary supplements to micronutrient intake and therefore providing better estimates of the micronutrient intake.

A possible limitation of the current study is the exclusion of certain age groups, such as infants and toddlers (between 0 and 2 years old) and elderly people (adults older than 64 years). The proportion of this elder population group in Belgium is considerable and increasing each year [52]. Furthermore, data on food consumption of the elderly are of particular interest as they are considered an important population group for dietary exposure assessments [6, 8]. The exclusion of these population groups was mainly due to logistic reasons, e.g. elderly require a particular methodology for the collection of food consumption information due to possible cognitive decline and it is often difficult to obtain an acceptable response rate, especially if institutionalised subjects are excluded [8].

Although great efforts were made to select a representative sample of the Belgian population, the participation rate of the BNFCS2014 was only 37 % (number of full participants/(number of eligible AND unresolved participants) [6]). The participation rate of the previous survey of 2004 was calculated as the ratio between the number of full participants and the number of individuals who met the selection criteria, and remained quite stable between 2004 (42 %) and 2014 (43 %). This low participation rate may introduce a sampling bias, but the distribution of the non-response across the sample has to be investigated further. However, to ensure representativeness of the Belgian population and reduce possible bias, weighting factors based on the probability of inclusion will be used during data analyses.

Another possible limitation is the fact that no biological samples (e.g. blood or urine) were collected in the present survey. For example, the evaluation of sodium intake in the population should preferably be estimated using 24-h urine collections as intake estimates based on 24-h recalls and FFQs strongly underestimate the intake of sodium [53]. There is an increasing interest in the use of dietary biomarkers as they are able to objectively assess dietary intake/status without the bias of self-reported dietary intake errors and can also overcome the problem of intra-individual diet variability. Moreover, dietary biomarkers can be used to validate self-reported intake results and evaluate nutrient intake when food composition tables are inadequate [54]. The main reason for not including biological samples was the considerable extra burden in terms of logistics, budget and practical consequences (e.g. the increase of response burden).

The results of the first national food consumption survey in 2004 have contributed to nutrition and food safety policy in Belgium. The previous survey revealed that food and dietary macronutrient intakes differed substantially from guidelines [12, 13]. As a result, the government started a campaign to improve dietary habits: the Belgian National Food and Health Plan (2005–2010) (BNFHP) [43]. The results of the first food consumption survey were also considered in the reformulation of nutritional recommendations by the Superior Health Council and food-based dietary guidelines (e.g. the active food triangle) for the Belgian population. In addition, several exposure assessments have been performed in the Belgian population using the national food consumption data of 2004 in combination with maximum permitted usage levels or actual usage levels of certain food additives (e.g. benzoic acid, sulphites, artificial sweeteners) [55–58]. These assessments are required by all member states of the European Union and serve as a base for legislative amendments if intake concerns are identified.

Although regular monitoring of dietary intake is crucial to evaluate time trends and policies on nutrition and food safety, no further monitoring of the Belgian food consumption was done between 2004 and 2014, demonstrating the importance of the organisation of a second Belgian food consumption survey in 2014–2015.

The BNFCS2014 will provide valuable data for the follow-up and evaluation of the BNFHP (e.g. has the consumption of fruit and vegetables increased, is there a more moderate and better quality fat intake, etc.). In the framework of other corrective dietary strategies, such as correcting the mild iodine deficiency in Belgium, data on the prevalence of use of iodised salt in households and habitual intake of iodine through foods and food supplements by the Belgian population in 2014 can be used for evaluating the effectiveness of these strategies. The BNFCS2014 will provide other useful and unique data such as the actual measured estimates of the prevalence of overweight and obesity in the Belgian population from 3 to 64 years old. The data of the BNFCS2014 will also be made available to external users for scientific research purposes. Today, exposure assessments still use the consumption data originating from 2004, while eating habits have presumably changed over the years. Moreover, currently exposure assessments cannot be performed for children because there are no appropriate consumption data for the Belgian population below the age of 15 years. This underlines again the importance of the BNFCS2014 data for re-evaluating exposure assessments, including children and adolescents.

The organisation of a (semi-) continuous monitoring system in Belgium would provide valuable information to regularly evaluate the dietary intake of the general Belgian population. This will allow the measurement of time trends and the development of effective policies on nutrition and food safety. Specific target groups such as elderly, infants and toddlers (which are important population groups for dietary exposure), would need separate complementary food consumption surveys as they require adapted approaches for the collection of food consumption information.

The final reporting is foreseen to be distributed in four separate reports: (1) eating habits, opinion on nutrition policies and anthropometry; (3) food safety; (2) physical activity and sedentary behaviour and (4) food, dietary supplement and nutrient intake (including the comparison with food-based dietary guidelines and nutritional recommendations by the Superior Health Council of Belgium). The first report can be expected at the end of 2015 and the last report in the course of 2016. The reports will be made available on the institutional website (https://fcs.wiv-isp.be).

## Conclusion

The main objective of the BNFCS2014 is to evaluate the habitual food, energy and nutrient intake in the Belgian population and to compare these with recommendations from the national dietary guidelines. A second objective is to monitor eating habits and food safety aspects of the food consumption in Belgium. The results of this dietary monitoring survey, together with the information on the level of physical activity, may underpin future nutrition, food safety and physical activity policies at national and European level (as part of the EU Menu project coordinated by EFSA).

## Abbreviations

PSU: Primary sampling unit
BMI: body mass index
BMR: basal metabolic rate
BNFCS2014: Belgian National Food Consumption Survey 2014
BNFHP: Belgian National Food and Health Plan (2005–2010)
DALYs: disability-adjusted life years
EFCOSUM: European Food Consumption Survey Method
EFCOVAL: European Food Consumption Validation
EFSA: European Food Safety Authority
EI: energy intake
EPIC: European Prospective Investigation into Cancer and Nutrition
FCS: food consumption survey
FFQ: food frequency questionnaire
IARC: International Agency for Research on Cancer
IPAQ: International Physical Activity Questionnaire
MVPA: moderate-to-vigorous physical activity
NCDs: non-communicable diseases
NPR: national population register
PA: physical activity
SB: sedentary behaviour
SPADE: Statistical Program to Assess Dietary Exposure
